# UNIMAGINED FUTURES: THE PARADOX OF FAMILISM AND ELDERCARE AMONG AGING LATINOS IN THE CHICAGOLAND AREA

**DOI:** 10.1093/geroni/igz038.2624

**Published:** 2019-11-08

**Authors:** Melissa Howe, Alexis Howard, Wendy Hsieh, Lissette M Piedra

**Affiliations:** 1 NORC at the University of Chicago, Chicago, Illinois, United States; 2 University of Illinois at Urbana-Champaign, Urbana, Illinois, United States

## Abstract

Scholars of gerontology highlight the ways aging varies cross-culturally. Whereas North Americans tend to describe “successful aging” as the maintenance of social and physical independence, Latin Americans tend to view aging as a natural process of social transition. In this study, we conducted a content analysis of nine focus groups (N =101) and 20 interviews with Latino older adults in the Chicagoland area to examine how they characterize successful aging and view the health declines that accompany aging. We found that Latino older adults often used rhetoric associated with “successful aging,” which tended to emphasize the maintenance of independence and physical functioning. Even immigrant respondents employed this language, suggesting that descriptions of “good old age,” may be more culturally transferable than previously thought. At the same time, the cultural values of respeto and familismo also emerged. Regardless of the participant’s nativity status, centrality of family and the importance of respect represented constant sources of support. Still, adherence to these values came with considerable drawbacks for those intensely focused on self-sacrifice for the sake of their families. Taken together, “successful old age” was defined by the participants as one in which a person maintains physical independence in the context of an interdependent, kin-focused, social life. This paradoxical combination of valuing independence and familial interdependence produced a number of benefits and challenges for Latino adults as they transitioned into to older adulthood.

